# Development of 3D Printed Multi-Layered Orodispersible Films with Porous Structure Applicable as a Substrate for Inkjet Printing

**DOI:** 10.3390/pharmaceutics15020714

**Published:** 2023-02-20

**Authors:** Jan Elbl, Martin Veselý, Dagmar Blaháčková, Jaroslav Ondruš, Pavel Kulich, Eliška Mašková, Josef Mašek, Jan Gajdziok

**Affiliations:** 1 Department of Pharmaceutical Technology, Faculty of Pharmacy, Masaryk University Brno, Palackého tř. 1946/1, 612 42 Brno, Czech Republic; 2 Department of Pharmacology and Toxicology, Veterinary Research Institute, Hudcova 70, 621 00 Brno, Czech Republic

**Keywords:** oral films, fast dissolving films, porous films, 3D print, inkjet print, individualized therapy

## Abstract

The direct tailoring of the size, composition, or number of layers belongs to the advantages of 3D printing employment in producing orodispersible films (ODFs) compared to the frequently utilized solvent casting method. This study aimed to produce porous ODFs as a substrate for medicated ink deposited by a 2D printer. The innovative semi-solid extrusion 3D printing method was employed to produce multilayered ODFs, where the bottom layer assures the mechanical properties. In contrast, the top layer provides a porous structure for ink entrapment. Hydroxypropyl methylcellulose and polyvinyl alcohol were utilized as film-forming polymers, glycerol as a plasticizer, and sodium starch glycolate as a disintegrant in the bottom matrix. Several porogen agents (Aeroperl^®^ 300, Fujisil^®^, Syloid^®^ 244 FP, Syloid^®^ XDP 3050, Neusilin^®^ S2, Neusilin^®^ US2, and Neusilin^®^ UFL2) acted as porosity enhancers in the two types of top layer. ODFs with satisfactory disintegration time were prepared. The correlation between the porogen content and the mechanical properties was proved. A porous ODF structure was detected in most samples and linked to the porogen content. SSE 3D printing represents a promising preparation method for the production of porous ODFs as substrates for subsequent drug deposition by 2D printing, avoiding the difficulties arising in casting or printing medicated ODFs directly.

## 1. Introduction

The most beneficial aspect of 3D printing in the preparation of dosage forms lies in the possibility of defining the exact drug dose to be printed, one which is specifically tailored to the individual patient and based on variables such as the patient’s age, sex, genetic makeup, and others. Three-dimensional printing also enables printing other than the established drug combinations within one application form. The most significant impact is to be perceived within medicines with a narrow therapeutic window and variable pharmacokinetics. This plays a crucial role in treating pediatric, geriatric, or polymorbidic patients, whose pharmacokinetics may significantly differ from those of the average population. The 3D printing of drugs thus presents a more individualized therapeutical approach [1,2,3,4,5]. 

Orodispersible films (ODFs) represent one of the most suitable dosage forms that can be 3D printed. ODFs could be defined as a thin dosage form intended for oral administration, with rapid disintegration or dissolving in the oral cavity [6]. ODFs can improve patient compliance as no additional liquid is required for administration. Moreover, patients having swallowing issues, such as pediatric, geriatric, or bed-prone patients, could benefit from this application form [7,8]. ODFs are formed from hydrophilic polymers which dissolve or disintegrate rapidly in the hydrophilic environment of the designated application site (tongue or buccal lining) [9,10]. A part of the released drug is absorbed via the oral cavity, pharynx, and esophagus into the systemic circulation. The hepatic first-pass effect is thereby partially evaded, which is one of the reasons why the onset of action is rapid and strong [11,12]. Initially, ODFs were developed as over-the-counter drugs (OTC). Currently, there is a significant number of prescribed-only ODFs [13].

The most common technique for ODF preparation is a solvent casting method, in which the active substance is a part of the film-forming dispersion, which is cast and subsequently dried [14,15]. The active substance could be degraded by the drying process or because of the high shear force arising during the dispersion mixing process [16,17]. Another issue in solvent casting preparation is entrapment of air bubbles within the film-forming dispersion during the mixing, caused by inappropriate dispersion viscosity, which leads to undesirable film heterogeneity [12]. Solvent casting produces one large film, which needs to be cut into several smaller films with the defined size. Punching or cutting the film can lead to uneven splitting or damage the film structure [18,19]. Film properties might also be affected by the employed drug. Careful selection of film-forming agents and drug combinations is therefore necessary.

The 3D printing of ODFs represents one possibility for avoiding solvent casting technique limitations. One of the most suitable methods is semi-solid extrusion (SSE) due to the relatively low temperature used in the process. Thermolabile materials could therefore be utilized. A defined number of single films of specific proportions may be printed; hence, the risk of uneven split or of damaging the film during the cutting process is diminished. Printing a definite number of layers with variable composition is also possible (drug combinations, modified release kinetics, etc.) [18,19]. 

The innovative approach of the 3D printing of blank ODFs and the subsequent API deposition by 2D printing on its surface can overcome the challenges mentioned above. Moreover, the 2D printing of API on the readily available substrate could shorten the preparation times of the drug dosage forms at point-of-care pharmacies, and it is especially suited for dosing low-dose APIs with a narrow therapeutic index since the dosing is more precise when compared to FDM or SSE 3D printing [16,17]. Inkjet printing or flexographic printing can be successfully exploited in this manner [1,16,17,20]. 

The blank ODF (representing the drug carrier and substrate for 2D printing) should be made of edible materials suitable for oral application [21]. A certain level of ODF mechanical strength and flexibility is desirable due to the frequent manipulation of the films. The interaction of the medicated ink and the ODF material should also be considered, as an inappropriate combination could result in film dissolvement or undesirable API crystallization. A sufficient level of film porosity is required as ODFs must be able to absorb even higher amounts of 2D printed medicines. Moreover, the medicated ink must be captured effectively on the film surface. Otherwise, ink leaking may result in the ODF’s untimely dissolution and disintegration. However, since the API is captured on the surface it does not interact with the film matrix, and the effect of API loading on the film’s mechanical properties is reduced [21,22,23]. 

In addition to better absorption properties, a higher porosity level protects the ODF from protuberance and hole formation upon ink printing. API crystallization might be less frequent on the porous surface [14,24]. Porogen addition is one way to increase ODF porosity. It was reported that porogen addition results in higher porosity and, therefore, a higher amount of ink being absorbed in the ODF structure [1,20]. In this regard, silicas (such as Aeroperl^®^ 300) are one of the most utilized porogen agents. Several studies have reported its beneficial impact on ODF porosity [20]. 

This study aims to produce porous unmedicated ODFs capable of absorbing medicated ink on their surface and structure. The need to develop porous, fast-disintegrating films with suitable mechanical properties has been mentioned in several studies [19,22]. Many studies focused on the solvent casting method, but to our knowledge, the number of works focusing on the possibility of using the 3D printing SSE method is relatively limited [17,20,25]. Such a preparation method with in-process drying was already successfully conducted, and the conclusions of the previous studies showed promising results [26,27]. This study is focused on the process feasibility, mechanical properties, disintegration time, and porosity of the prepared ODFs. For this purpose, the novel two-layer film model was designed, where the base layer is supposed to guarantee mechanical durability and flexibility. In contrast, the top layer containing the selected porogen agent provides a porous surface and structure to absorb and retain medicated ink printed by complementary 2D printing. 

## 2. Materials and Methods

### 2.1. Materials

Polyvinyl alcohol (Mowiol^®^ 4–88; PVA) was purchased from Sigma Aldrich (Taufkirchen, Germany), and hydroxypropyl methylcellulose (Pharmacoat^®^ 606; HPMC) was acquired from Shin Etsu (Tokyo, Japan), both acting as film-forming polymers. Glycerol (Gly), used as a plasticizer, was obtained from Dr. Kulich Pharma (Hradec Králové, Czech Republic). Sodium starch glycolate (Explotab^®^; Ex), used as a disintegration agent, was purchased from JRS Pharma (Rosenberg, Germany). Ethanol 96% (Et), used as a dispersing agent of sodium starch glycolate, was purchased from Penta (Praha, Czech Republic). Xanthan (X), used as a viscosity-altering agent, was purchased from Sigma Aldrich (Taufkirchen, Germany). The following porogens were used: silicon dioxide (Aeroperl^®^ 300; A), purchased from Evonik (Essen, Germany); (Fujisil^®^; F), purchased from Fuji Chemical Industries Co., Ltd. (Tokyo, Japan); (Syloid^®^ 244 FP; SFP) and (Syloid^®^ XDP 3050; SX), both purchased from Grace GmbH (Worms, Germany); and magnesium aluminometasilicate (Neusilin^®^ S2; NS), (Neusilin^®^ US2; NUS), and (Neusilin^®^ UFL2; NUFL), purchased from Fuji Chemical Industries Co., Ltd. (Tokyo, Japan). Purified water (W) with a quality responding to Ph. Eur. was used.

### 2.2. Print Dispersion Preparation and Viscosity Evaluation

The composition of the used print dispersion is presented in Table 1. For matrix dispersion, separate 12.5% stock solutions of PVA and HPMC were prepared by mixing the respective polymers with purified water under constant magnetic stirring for 12 h. These solutions were then combined in relevant ratios. Gly was added afterwards, followed by the addition of the dispersion of Ex in Et (1:1), both at a slow pace under continual magnetic stirring. The dispersion was left at a constant stirring for 60 min until homogenized. This print dispersion was used to print the bottom film layer.

For the top layer, two different compositions were utilized. The type A composition samples were prepared by slowly adding a certain porogen amount to the bottom layer composition under constant stirring, replacing the respective part of W. Two bottom layers and three top layers were printed. The type A composition samples are marked by the used porogen abbreviation plus its concentration. Three different concentrations of each porogen agent and seven different porogens accounted for 21 samples of the type A composition.

The type B composition was created by adding 12.5% PVA stock solution to the xanthan gum stock solution. This mixture was left until homogenized, and the porogen addition under constant stirring was the final preparation step. Three bottom and two top layers were printed in the type B composition samples to compensate for the fact that the top layers contain only loosely bound porogen particles and cannot effectively add to the overall mechanical properties of the ODFs. The type B composition samples are marked by “X”, followed by the utilized porogen abbreviation plus its concentration within the sample. In this case, only two distinct concentrations of the respective porogen were used. The type B composition thus involves 14 samples.

To evaluate the viscosity of the print dispersion, a DV-II + Pro viscometer (AMETEK Brookfield, Middleboro, MA, USA) was employed. The measurements were conducted using a small sample volume (10 mL of sample) adapter and an SC4-27 spindle set to rotate at 200 RPM (shear rate 186 s^−1^). The temperature was kept in the 25 ± 0.1 °C range using an external water bath. Each sample was measured once.

### 2.3. SSE 3D Printing

**Figure 1:**
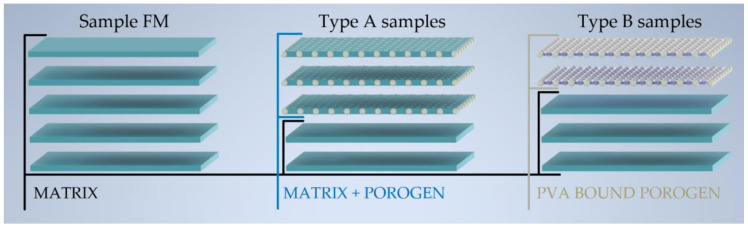
Graphical representation of layer composition in different sample types.

Digital models of the films were prepared in Autodesk Inventor 2022 (Autodesk, Portland, OR, USA) CAD software. Each batch consisted of 25 rectangular films of 20 × 30 mm base dimensions in the bottom layers and 19.16 × 29.16 mm in the top layers, with the thickness of each layer being set to 0.02 mm. For matrix optimization, 25 rectangular films of 20 × 30 mm base and 0.1 mm height (5 × 20 µm layer) were printed. These designs were saved as a stereolithographic file (.stl) and exported to the Slic3r PE 1.33.8 (Prusa Research ltd., Praha, Czech Republic). Figure 1 shows the setup of the layers in different samples for further clarification.

This study utilized an in-house SSE printer with 4 independent extruders designed as linear syringe pumps. Syringes of 50 mL (with an internal diameter of 28 mm) were used to supply the material through a 50 cm tubing to an 18 G stainless needle tip (0.84 mm dia.). Films were printed on 90 µm thick polyester masking tape (Lepíky ltd., Praha, Czech Republic) laid on a 2 mm glass sheet.

The print settings were as follows: bed temperature 70 °C, print speed 50 mm/s, extrusion width 0.84 mm, 1 perimeter, and 100% rectilinear pattern infill density. The films were kept on the printing bed for 10 min at 70 °C after finishing the print, to provide sufficient drying.

### 2.4. Weight

All 25 films were weighed using KERN 220-4N analytical scales (Gottl. KERN & Sohn GmbH, Balingen, Germany). The obtained results are presented as a mean value ± SD.

### 2.5. Thickness

Data on the film thickness were obtained in the whole batch (*n* = 25), using the coating thickness gauge Elcometer 456 (Elcometer Limited, Manchester, UK). The thickness was measured at four film corners and in the middle, resulting in five different measurement locations within a single film [26]. The results are presented as a mean value ± SD. The obtained data were utilized to further recalculate the data for disintegration time to the uniform thickness of 100 µm and to calculate the samples’ tensile strength.

### 2.6. Mechanical Properties

Texture analysis was used to evaluate the mechanical properties of the printed ODFs. For this purpose, a CT3 Texture Analyzer (AMETEK Brookfield, Middleboro, MA, USA) equipped with a 4.5 kg load cell and controlled by TexturePro CT software (AMETEK Brookfield, Middleboro, MA, USA) was used [26]. For both the tensile and the puncture testing, five different samples were randomly selected. All values are presented as a mean value ± SD.

For tensile testing, the films were held between two clamps of the TA-DGA probe, positioned at an initial distance of 1 cm. The lower clamp remained stationary while the upper clamp constantly moved upwards at a rate of 0.5 mm/s, pulling the film apart until breakage. The measured data included the force and work done during the process and the film elongation at the time of tearing. The calculation of the tensile strength was performed by dividing the tensile force (TF) at which the breakage occurred by the film’s cross-sectional area (mm^2^). All the data, except TF, were also recalculated to the 100 µm thickness of the film for further comparison [26].

For puncture testing, the films were fixed in the JIG TA-CJ holder, and the TA39 cylindrical probe (2 mm diameter, probe motion speed 0.5 mm/s) was used to penetrate the film. The measured data included the maximum force required to puncture the film, its deformation, and the work done in the process. All the obtained data were also recalculated to the 100 µm film thickness [26].

### 2.7. Disintegration Time

A modified disintegration tester with film holder clamps was used to evaluate the disintegration time. Five randomly selected ODFs were selected for the measurement. The 3 g weight was attached to the bottom edge of the ODFs, representing the minimal force applied by the human tongue [28]. The upper film edge was magnetically pinned to the tester. To simulate the oral cavity environment, the test vessel was filled with 600 mL phosphate buffer with a pH of 6.8. The testing temperature was set to 37 °C. The samples were cyclically immersed and withdrawn from the buffer (30 cycles per minute), and the disintegration time was visually confirmed as the clamped weight dropped [26]. The results are presented as a mean value ± SD.

### 2.8. Micro-CT

Micro-CT analysis was conducted on a Bruker micro-CT SkyScan 1276 (Bruker, Kontich, Belgium). The films were scanned at 40 kV, 100 µA, on the 12 mm diameter scanning bed in the step-and-shoot mode with 0.2 rotation steps and 1032 projections. The camera was set to 4K resolution and 2 × 2 binning. No filter was used. The pixel size was 2.8 µm. The reconstruction of the backward projection datasets of all the ticks was performed by Insta-Recon software (Bruker microCT, Kontich, Belgium). The misalignment compensation and region of interest were selected manually for every sample. The ring artifact reduction was set to 10 and the values of the dynamic image range to 0.00–0.10. CTanalyser software (Bruker microCT, Kontich, Belgium) performed the post-processing adjustments and porosity analyses. CTVox (Bruker microCT, Kontich, Belgium) was used for the 3D visualization of the reconstructed datasets.

### 2.9. Scanning Electron Microscopy

The samples were put on carbon tape attached to the aluminum holder. They were observed under a scanning electron microscope Hitachi SU 8010 (Hitachi High Technologies, Minato, Japan) at a magnification of 50–1100× (at 10 kV, SE detector, and the working distance of 8 mm).

## 3. Results and Discussions

### 3.1. SSE 3D Printing, Viscosity Evaluation

In initial testing, the matrix composition was optimized (non-published data). It was found that the ratio of 1.5% HPMC, 5% PVA, 5% Gly, and 2.5% Ex with 2.5% Et led to the production of non-fragile films that were easily removable from the print bed. As a next step, selected porogens in various concentrations were added directly to this matrix composition to test whether printing spatially non-differentiated ODFs of sufficient porosity was possible. However, the obtained films were fragile and almost impossible to remove from the printing bed. It was concluded that porogen particles impaired the formation of a suitable film matrix, probably due to the high specific surface area available for the binding of the film polymers.

Based on these findings, multi-layered ODFs were printed, with each layer performing a distinct task. The bottom layer (with its composition being the same as a porogen-free matrix) would contribute to the suitable mechanical properties, and the top layer (containing porogen) would ensure adequate porosity.

The type A composition samples containing A, F, NS, NUS, and the middle and highest NUFL concentrations (1% and 1.5%) exhibited suitable mechanical properties and were easily removable from the printing bed. All the ODFs of type A were printed with two bottom layers and three top layers, demonstrating a convenient ratio in terms of the handling properties of ODFs. The samples with SFP, SX, and the lowest NUFL concentration (0.5%) exhibited imperfect structures with several ruptures occurring upon removal from the printing bed. However, the type A composition samples also showed low porosity. It was concluded that film-forming excipients tend to envelop particles of porogen, diminishing the porosity (yet not enough to assure good film properties, as was evident from the initial tests).

**Figure 2:**
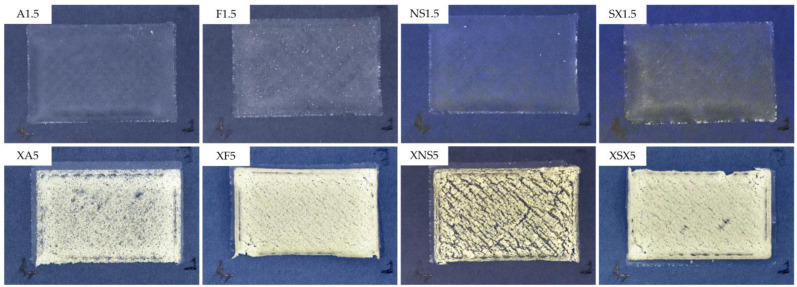
Appearance of selected samples of type A and type B ODFs.

Therefore, the type B composition ODFs were printed. The top layer consisted of 1% PVA, which could ensure sufficient binding of the individual porogen particles while not fully enveloping them so that structural porosity would be retained. The addition of xanthan gum to the top layer dispersion in the stated concentration (Table 1) provided better kinetic stability in order to prevent sedimentation of the porogen in the syringe throughout the printing process. The selected ODFs are depicted in Figure 2.

All the ODFs of the type B composition were printed with three bottom layers and two top layers to accommodate the expected decrease in mechanical properties stemming from the fact that the top layers consisted of only loosely bound porogen particles and to reduce the effect of porogen migration into the bottom layers, which negatively influenced the mechanical properties of the type A ODFs.

The type B composition samples containing A, SX, and a lower concentration of NS (2.5%) exhibited satisfactory mechanical characteristics and flexibility properties. The films containing F, a lower concentration of NUS, NUFL, and SX (2.5%), and a higher concentration of NS (5%) demonstrated imperfections such as minor ruptures upon removal from the print bed. The ODFs with a higher concentration of NUS, NUFL, and SFP (5%) exhibited unsatisfactory properties. Significant ruptures occurred upon removal from the print bed. It was impossible to remove the films without damaging them. Hence, these samples were not evaluated further.

All the print dispersions showed viscosity within the acceptable range usable for this printer (Table 2), as the viscosity should not exceed 3000 cP (unpublished in-house limit). In general, the type A samples exhibited higher viscosities, although they contained less porogen than the type B dispersions. This could be attributed to the overall composition of the matrix used in the type A dispersions. There was also a clear trend of viscosity increase with the porogen content in individual sets of both the A type and the B type dispersions.

### 3.2. Weight

Uniformity of weight is one of the crucial aspects of ODFs, due to its correlation with dose uniformity and the mechanical properties of films (the content of residual solvent). As ODFs, in this case, are unmedicated, weight uniformity is regarded mainly as a critical indicator of printing quality. Low weight variability was achieved (RSD_max_ = 4.83; XF2.5) in all the samples (Table 2), hinting at good printing quality and the repeatability of the process. A slight difference in weight was observed between the samples with different concentrations of the respective porogen.

No correlation between the porogen concentration and the weight was found within the type A composition but could be observed within the type B composition. This could be attributed to both the higher porogen content and the higher relative content of porogen in the type B composition when related to the other excipients, as well as to a broader range of porogen content in the type B composition samples (2.5% to 5% in type B vs. 0.5% to 1% to 1.5% in type A). The lower average weight of the type B composition samples is explained simply by the lesser amount of solid material in the printing of the type B top layer.

### 3.3. Thickness

The typical ODF thickness ranges between 10 µm and 100 µm [15]. However, examples of thicker ODFs have also been reported [9]. The average thickness of all the samples of printed ODFs was between 77.07 and 149.87 µm and therefore within the suggested limits. The variability in thickness (Table 2) was generally higher for the type A composition samples. This was caused by the uneven spreading of the material due to the higher viscosity of the top layer composition, as proven by the viscosity measurement of the print dispersion (Table 2); this is further discussed in the section concerning SEM imaging.

A drop in thickness was observed when comparing the ODFs of the type A and type B compositions (Table 2). The lower thickness in the type B composition ODFs could be attributed to the smaller amount of material utilized in the top layer. No correlation between the porogen concentration and the thickness of the ODFs was observed within the type A composition. Conversely, this correlation was achieved in the type B composition as the higher porogen content resulted in thicker ODFs.

### 3.4. Mechanical Properties

Sufficient mechanical properties are essential for ODFs as possible ruptures in the film structure could result in deterioration of the handling properties and in application issues, limiting patient compliance [15,28]. As no pharmacopeial limits are set for these properties, the only recommended ranges are based on the literature which has reviewed the already marketed films. According to Preis et al., who evaluated the mechanical properties of the marketed ODFs and buccal films, puncture strength is usually found within the range of 0.08–0.40 N.mm^−2^, with the respective elongation to puncture being between 1.03 and 6.54% [29]. As for tensile properties, Gupta et al. reported an optimum tensile strength of at least 2 N.mm^−2^ and an elongation to break of >10% [30]. The results of the tensile testing are summarized in Table 3.

Generally, lower tensile strength (TS) was observed in the type B composition ODFs compared to the type A. This is because the type A samples’ top layers contain more film-forming polymers contributing to the overall TS. Conversely, the type B samples’ top layer consists mainly of porogen, which does not contribute to mechanical strength, but adds to the thickness, respectively the cross-sectional area of the films, lowering the TS in the final effect. This conclusion complies with those of other studies [11]. The elongation at break found in all the samples was acceptable when compared to the values reported by Gupta et al. [30]. The type A composition ODF samples with the highest amount of A, NS, and NUS (1.5%) possessed the highest TS. A relation between the TS and the porogen content was not observed, as higher porogen content in the ODFs did not necessarily result in a lowered TS (Table 3). These outcomes are not in full accordance with the available literature as higher porogen content usually leads to the lowered TS of the ODFs [11,25]. 

The type B composition ODF samples with the lower amount of A, NS, and SX (2.5%) possessed the highest TS (Table 3), falling into the region of TS outlined by Gupta et al. as acceptable [30]. The other samples (except XF5) exhibited TS in the range between 1 and 2 N/mm^2^ and could be considered acceptable if the effect of the porogenous top layer on the relative TS is accounted for.

The ODFs with lower porogen particles represented the samples with higher TS. This complies with the findings of Takeuchi et al., reported that a higher porogen amount leads to ODFs with a lower mechanical strength [25]. 

Similarly, as in the tensile testing, the type B composition samples exhibited lower puncture strength (PS) (Table 4). This drop could be attributed to the higher porogen content in the type B composition samples. In other studies, this conclusion was also confirmed [11]. All the samples exhibited PS values within the range of 0.08–0.40 N/mm^2^, as specified by Preis et al. [29].

The type A composition ODF samples containing A and NUS exhibited the highest PS. In most cases, the higher porogen concentration decreased the PS, except for the NUFL samples, which showed almost identical PS and SX samples where the PS increased with porogen content. This follows the available literature [11,25]. The elasticity represented by elongation to puncture also decreased along with the porogen content in most samples. 

The type B composition ODF sample XSX2.5 represented the ODF with the highest puncture strength (Table 4). The detrimental effect of the porogen content on the ODF mechanical properties reported by Takeuchi et al. was confirmed by the decreasing PS and elongation to puncture values only in the XA and XNS samples [25]. 

Compared to the FM matrix sample, there is a noticeable effect of porogen loading on the matrix puncture strength and elongation to break properties, yielding less flexible films that are more prone to breakage. In agreement with the tensile properties, the decrease in puncture properties is more significant than expected, considering the samples’ composition, pointing at porogen migration throughout the printing process.

When compared to the matrix sample FM, all the samples exhibited lower TS. The drop in TS was greater than expected, considering that the FM sample consisted of five layers of matrix composition. In contrast, the type A samples consisted of only two such layers and three layers with a porogen, and the type B samples comprised three matrix layers and two layers of the weakly bound porogen. This indicates that the porogen migrates to the already printed layers (when those are re-dissolved by newly deposited dispersion) and reduces their contribution to the overall TS. 

### 3.5. Disintegration Time

There is no standardized test for determining ODF disintegration time (DT). Moreover, no official time limit has been stated in Ph. Eur. or by the FDA. Most often in ODF evaluation, the DT limits concerning orodispersible tablets (ODT) defined as being 30 s (FDA) or 180 s (Ph. Eur.) are considered sufficient [31,32,33]. In this study, the stricter FDA limit for ODT was explicitly considered.

All the printed ODFs complied with the time limit of 30 s outlined by the FDA for ODT (Table 5). Sufficient DT was achieved in both the A type and the B type compositions despite the high DT variability observed. 

The type A composition ODFs showed no correlation between the DT and the porogen content. On the other hand, such an effect was confirmed in all the comparable samples of the type B composition ODFs, except the samples with NS content. A decrease in DT was found in the type B composition ODFs compared to the type A composition samples. This is caused by the higher amount of porogen agent in the type B samples and by the presence of film-forming polymers in the top layers of the type A samples. These results follow other studies proving that a higher content of porogens or insoluble fillers shortens the DT of ODFs [11,25,34]. 

### 3.6. Micro-CT

A certain level of ODF porosity is essential to embed the required amount of drug that could be adsorbed on the film surface and into its structure [35]. 

**Figure 3:**
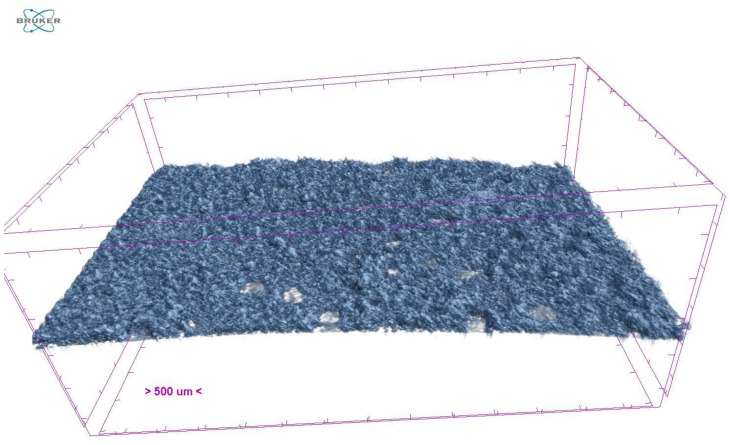
Micro-CT reconstruction of XF5 sample.

Only samples with the highest amount of porogen particles were evaluated as, according to the available literature, the highest impact on ODF porosity is linked more to higher than lower porogen content [20]. Promising results were observed in the type B composition ODFs. Most of them achieved higher porosity levels than the FM sample (Table 5 and Figure 3). Conversely, the type A composition ODFs possessed a lower porosity level than the FM (except sample F1.5) (Table 5). The higher porosity levels found in the type B composition ODFs could be attributed to the different composition of the top layer and the higher porogen content. Increasing the ODF porosity level by adding more porogen into its structure has been repeatably reported [20].

### 3.7. Scanning Electron Microscopy

**Figure 4:**
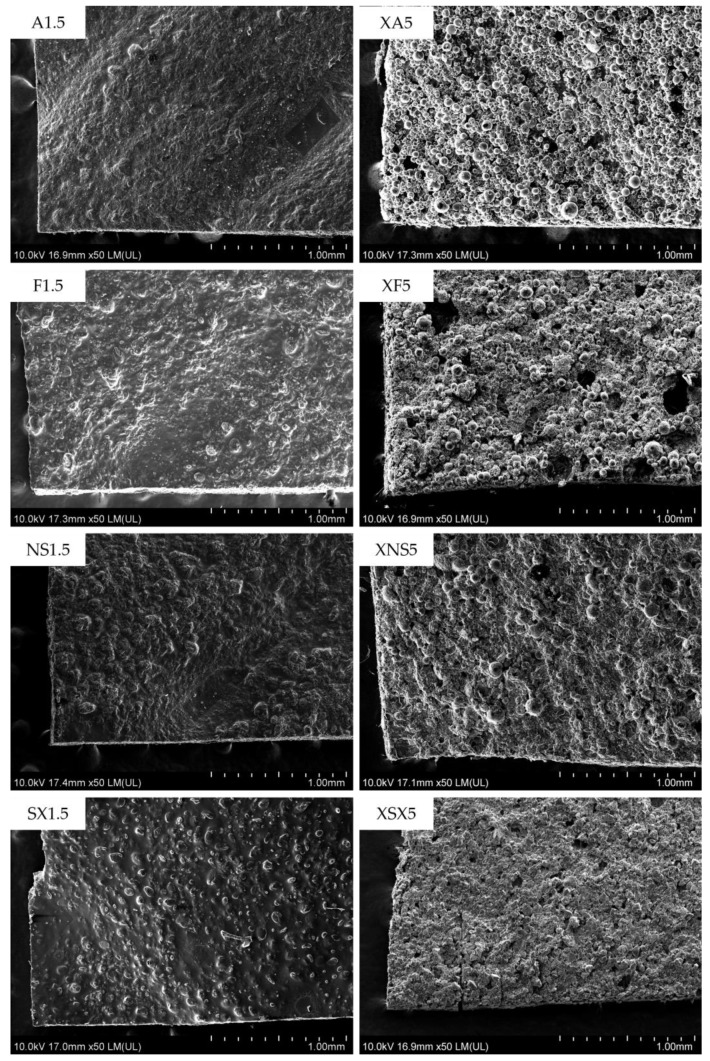
SEM images of selected ODFs: **left**—type A samples; **right**—type B samples.

Only the samples of the type A and type B compositions containing the highest amount of A, F, and NS porogen agents were investigated by scanning electron microscopy (SEM). The obtained images confirm the conclusions made by micro-CT testing (Table 5). The increased porosity is evident within the type B composition samples (Figure 4) since the top layer is formed by discrete porogen particles bound only by a small amount of PVA. In the type A ODFs, only partially protruding particles are seen embedded into the matrix of the films regardless of the porogen type. Such a structure would not allow a significant amount of ink to be absorbed into the film through subsequent 2D printing drug deposition.

The ODFs of both composition types possess a certain level of unevenness. For the type A composition, the uneven material deposition could be attributed to the higher viscosity (Table 2) and inadequate rheological properties of the print dispersion containing a high amount of film-forming polymers and porogen. The individual extrusion strands are not overlapped properly and form ridges and depressions in the topmost layer. This is the main reason for the high thickness variability in the type A composition samples.

The small, localized holes in the top layer of the type B samples may theoretically be formed due to the momentary block of the print needle by larger aggregates of the porogen. However, these aggregates would be relatively quickly pushed through the needle by a build-up of pressure since more severe blockage would result in a completely missing part of a printed layer and an unsatisfactory weight uniformity of the affected samples.

## 4. Conclusions

Multilayered SSE 3D printed ODFs with incorporated porogen were successfully prepared. All the ODFs met the criteria for weight variability and disintegration time (considering the usually applied FDA limit for the disintegration of ODT). Two novel strategies in porogen incorporation were used to tackle the balance between the mechanical properties and the porosity of the ODFs. Initially, the porogen was incorporated only in the top layers of an otherwise homogenous film matrix, yielding ODFs with good mechanical properties but low porosity. The second strategy, where the bottom matrix layers served as a backing for top layers consisting of porogen loosely bound by a small amount of PVA, yielded ODFs of sufficient mechanical strength and porosity.

In conclusion, SSE 3D printing is feasible for preparing multi-layer porous ODFs. Drug deposition by 2D printing on the blank film is a promising subsequent method for medicating ODFs.

## Figures and Tables

**Table 1:** Composition of print dispersions.

Composition Type	Sample	Excipient Concentration in Dispersion (wt%)							
		PVA	HPMC	Gly	Ex + Et	W	X	Porogen	No of Layers
Bottom layer matrix	FM	5	1.5	5	2.5 + 2.5	83.5	-	X	2 or 3
Type A top layer	*0.5	5	1.5	5	2.5 + 2.5	83.0	-	0.5	3
	*1	5	1.5	5	2.5 + 2.5	82.5	-	1.0	3
	*1.5	5	1.5	5	2.5 + 2.5	82.0	-	1.5	3
Type B top layer	X*2.5	1	-	-	-	96.0625	0.4375	2.5	2
	X*5	1	-	-	-	93.5750	0.4250	5.0	2

* Abbreviation of the used porogen.

**Table 2:** Viscosity of print dispersions and measurements of the weight and thickness of ODFs.

Composition Type	Sample	Viscosity (cP)	AVG Weight (mg)	RSD (%)	AVG Thickness (µm)	RSD (%)
MATRIX	FM	57.10	88.0 ± 3.80	4.32	124.11 ± 17.75	14.26
A	A0.5	67.25	83.8 ± 0.64	0.76	130.86 ± 30.70	23.46
	A1	96.50	86.1 ± 0.51	0.59	134.62 ± 30.50	22.66
	A1.5	118.00	85.7 ± 1.27	1.48	125.24 ± 27.00	21.56
	F0.5	98.25	80.6 ± 0.58	0.72	107.25 ± 17.00	15.85
	F1	108.50	80.9 ± 0.67	0.83	114.15 ± 17.19	15.06
	F1.5	116.80	83.9 ± 0.73	0.87	128.93 ± 22.78	17.67
	NS0.5	79.25	80.5 ± 0.66	0.82	110.04 ± 19.86	18.05
	NS1	112.80	83.8 ± 0.68	0.81	120.61 ± 19.67	16.31
	NS1.5	195.80	83.9 ± 0.73	0.87	115.78 ± 17.38	15.01
	NUS0.5	122.00	78.8 ± 1.73	2.19	107.87 ± 17.93	16.62
	NUS1	127.30	79.8 ± 0.73	0.92	131.77 ± 21.47	16.29
	NUS1.5	136.50	83.2 ± 2.62	3.15	149.87 ± 33.18	22.14
	NUFL0.5	109.50	86.4 ± 2.03	2.35	115.16 ± 19.21	16.68
	NUFL1	174.30	85.2 ± 2.01	2.36	115.26 ± 17.61	15.28
	NUFL1.5	216.80	91.1 ± 3.17	3.48	136.78 ± 23.21	16.97
	SFP0.5	76.75	86.3 ± 0.81	0.94	111.12 ± 14.06	12.65
	SFP1	124.30	90.0 ± 0.98	1.09	112.21 ± 21.30	18.98
	SFP1.5	187.50	90.4 ± 0.89	0.99	110.78 ± 18.08	16.32
	SX0.5	69.25	86.6 ± 1.10	1.27	102.92 ± 15.67	15.23
	SX1	89.25	86.9 ± 1.46	1.68	105.14 ± 10.73	10.21
	SX1.5	111.00	89.7 ± 1.46	1.63	127.05 ± 20.66	16.26
B	XA2.5	71.00	61.6 ± 0.68	1.11	77.34 ± 7.86	10.16
	XA5	95.00	63.1 ± 1.53	2.43	96.73 ± 10.98	11.35
	XF2.5	74.00	61.0 ± 2.95	4.83	82.91 ± 10.53	12.70
	XF5	105.30	66.5 ± 1.96	2.94	113.59 ± 13.97	12.30
	XNS2.5	76.75	62.4 ± 0.80	1.28	77.07 ± 10.38	13.47
	XNS5	84.75	66.3 ± 1.33	2.01	87.43 ± 11.86	13.57
	XNUS2.5	73.25	56.3 ± 0.83	1.47	79.92 ± 10.31	12.90
	XNUFL2.5	77.00	59.7 ± 0.46	0.77	78.26 ± 10.92	13.95
	XSFP2.5	88.50	62.4 ± 0.34	0.54	79.36 ± 15.13	19.06
	XSX2.5	67.25	61.9 ± 0.58	0.93	78.50 ± 8.81	11.22
	XSX5	94.25	67.6 ± 0.55	0.81	114.47 ± 16.17	14.13

**Table 3:** Results of tensile testing.

Composition Type	Sample	Tensile Strength (N/mm^2^)	SD (N/mm^2^)	Elongation at Break (%)	SD (%)
MATRIX	FM	7.07	0.24	45.80	10.56
A	A0.5	2.99	0.21	59.00	8.23
	A1	3.09	0.11	55.50	4.83
	A1.5	3.70	0.11	59.60	6.69
	F0.5	2.64	0.11	30.80	3.12
	F1	2.43	0.18	23.00	2.00
	F1.5	2.11	0.14	19.30	3.20
	NS0.5	3.50	0.07	50.70	5.10
	NS1	3.39	0.08	46.40	2.07
	NS1.5	3.94	0.13	46.40	6.84
	NUS0.5	3.97	0.12	52.20	6.36
	NUS1	3.25	0.14	37.80	3.21
	NUS1.5	2.95	0.14	35.10	4.59
	NUFL0.5	1.86	0.15	21.60	3.51
	NUFL1	2.00	0.16	21.00	3.18
	NUFL1.5	1.68	0.11	18.80	1.28
	SFP0.5	2.12	0.19	24.90	3.10
	SFP1	2.01	0.14	22.40	2.48
	SFP1.5	2.07	0.04	24.90	2.16
	SX0.5	1.65	0.02	29.30	0.90
	SX1	2.16	0.06	29.50	0.83
	SX1.5	1.79	0.01	24.70	1.46
B	XA2.5	2.33	0.09	17.10	1.87
	XA5	1.13	0.05	16.00	1.86
	XF2.5	1.56	0.10	11.70	1.32
	XF5	0.85	0.28	7.70	2.57
	XNS2.5	1.98	0.27	17.10	2.68
	XNS5	1.63	0.06	11.10	0.79
	XNUS2.5	1.27	0.07	12.50	0.28
	XNUFL2.5	1.76	0.27	12.80	2.73
	XSFP2.5	1.19	0.25	7.80	1.45
	XSX2.5	1.99	0.30	13.30	3.13
	XSX5	1.22	0.10	12.40	0.84

**Table 4:** Results of puncture testing.

Composition Type	Sample	Puncture Strength (N/mm^2^)	SD (N/mm^2^)	Elongation to Puncture (%)	SD (%)
MATRIX	FM	1.95	0.26	48.51	6.56
A	A0.5	1.09	0.07	21.34	1.16
	A1	0.89	0.06	16.51	0.55
	A1.5	1.05	0.08	17.16	0.81
	F0.5	0.6	0.03	9.69	0.44
	F1	0.52	0.06	8.05	0.44
	F1.5	0.48	0.05	6.61	0.36
	NS0.5	0.88	0.14	18.87	1.34
	NS1	0.64	0.01	9.84	0.35
	NS1.5	0.58	0.08	8.75	0.69
	NUS0.5	0.92	0.10	12.84	1.05
	NUS1	0.94	0.07	12.1	0.56
	NUS1.5	0.79	0.14	9.61	0.81
	NUFL0.5	0.33	0.04	6.23	0.21
	NUFL1	0.32	0.03	5.58	0.41
	NUFL1.5	0.33	0.03	5.46	0.16
	SFP0.5	0.49	0.03	8.96	0.35
	SFP1	0.39	0.03	6.99	0.23
	SFP1.5	0.36	0.01	7.25	0.27
	SX0.5	0.31	0.02	8.32	0.18
	SX1	0.38	0.02	7.91	0.24
	SX1.5	0.40	0.03	7.58	0.43
B	XA2.5	0.22	0.03	4.21	0.49
	XA5	0.13	0.01	3.91	0.45
	XF2.5	0.15	0.03	3.21	0.44
	XF5	0.16	0.03	3.62	0.33
	XNS2.5	0.22	0.02	4.68	0.30
	XNS5	0.18	0.01	3.30	0.24
	XNUS2.5	0.15	0.01	3.72	0.23
	XNUFL2.5	0.21	0.01	4.53	0.21
	XSFP2.5	0.14	0.05	2.94	0.65
	XSX2.5	0.25	0.02	4.63	0.28
	XSX5	0.25	0.03	5.18	0.40

**Table 5:** Results of DT and micro-CT porosity evaluation.

Composition Type	Sample	AVG DT (s)	AVG DT to 100 µm (s)	RSD (%)	Porosity
MATRIX	FM	17.90 ± 1.57	14.42 ± 1.26	8.75	8.7
A	A0.5	13.67 ± 0.80	10.45 ± 0.61	5.83	X
	A1	12.95 ± 0.47	9.62 ± 0.35	3.60	x
	A1.5	12.23 ± 1.25	9.76 ± 1.00	10.24	1.76
	F0.5	9.95 ± 1.53	9.27 ± 1.42	15.35	x
	F1	10.50 ± 1.20	9.20 ± 1.05	11.41	x
	F1.5	10.74 ± 1.14	8.33 ± 0.88	10.61	8.94
	NS0.5	11.98 ± 0.53	10.88 ± 0.48	4.39	x
	NS1	13.01 ± 0.65	10.79 ± 0.54	5.02	x
	NS1.5	12.08 ± 0.80	10.43 ± 0.69	6.60	1.29
	NUS0.5	9.82 ± 1.83	9.10 ± 1.70	18.69	x
	NUS1	11.10 ± 0.38	8.43 ± 0.29	3.46	x
	NUS1.5	9.25 ± 3.16	6.17 ± 2.11	34.17	2.24
	NUFL0.5	11.50 ± 0.14	9.99 ± 0.12	1.22	x
	NUFL1	11.33 ± 1.32	9.83 ± 1.15	11.66	x
	NUFL1.5	10.94 ± 1.53	8.00 ± 1.12	13.99	7.38
	SFP0.5	12.19 ± 0.66	10.97 ± 0.60	5.43	x
	SFP1	10.46 ± 1.09	9.33 ± 0.97	10.40	x
	SFP1.5	9.39 ± 0.42	8.48 ± 0.38	4.48	2.25
	SX0.5	9.76 ± 0.41	9.48 ± 0.40	4.25	x
	SX1	10.81 ± 0.88	10.28 ± 0.84	8.16	x
	SX1.5	10.37 ± 0.54	8.17 ± 0.43	5.22	3.18
B	XA2.5	5.57 ± 0.28	7.20 ± 0.36	4.96	x
	XA5	3.69 ± 0.09	3.82 ± 0.09	2.42	17.64
	XF2.5	4.61 ± 0.46	5.56 ± 0.56	10.04	x
	XF5	3.46 ± 0.49	3.05 ± 0.43	14.17	22.76
	XNS2.5	4.71 ± 0.46	6.12 ± 0.59	9.69	x
	XNS5	5.47 ± 0.58	6.26 ± 0.67	10.66	16.75
	XNUS2.5	3.55 ± 0.12	4.44 ± 0.15	3.31	10.36
	XNUFL2.5	5.31 ± 0.33	6.78 ± 0.42	6.13	6.11
	XSFP2.5	3.85 ± 0.51	4.86 ± 0.64	13.21	21.34
	XSX2.5	5.45 ± 0.48	6.95 ± 0.61	8.83	x
	XSX5	4.27 ± 0.31	3.73 ± 0.27	7.23	14.26

## Data Availability

Data are contained within the article.
